# The features and risk factors of thrombotic thrombocytopenic purpura in systemic lupus erythematosus

**DOI:** 10.1186/s13023-025-03741-0

**Published:** 2025-04-28

**Authors:** Hang Ma, Yujie He, Shanshan Li, Yingchao Yang, Liubin Huo, Tianfang Li

**Affiliations:** 1 Department of Rheumatology, The First Affiliated Hospital of Zhengzhou University, Zhengzhou, 450052 Henan China; 2 Peking University China-Japan Friendship school of clinical medicine, Beijing, 100029 China; 3 Department of Rheumatology, Key Myositis Laboratories, China-Japan Friendship Hospital, Beijing, China

**Keywords:** Thrombotic thrombocytopenic purpura, Systemic lupus erythematosus, Risk factor

## Abstract

**Objective:**

This study aimed to investigate clinical features and risk factors for the development of thrombotic thrombocytopenic purpura (TTP) in systemic lupus erythematosus (SLE) patients.

**Methods:**

A cohort of 32 SLE-TTP patients in the first affiliated hospital of Zhengzhou University from 2017 to 2023 were included, and 128 SLE patients without TTP admitted to the hospital during the same period were randomly selected as the control group. The demographic data, clinical and laboratory findings of these patients were statistically analyzed. Stepwise regression and logistic regression were used to identify the risk factors related to TTP development. The SLE-TTP patients were divided into two groups based on treatment outcomes, and the differences between the clinical data were compared between the two groups. Independent risks of short-term death in SLE-TTP patients were determined by logistic regression analysis.

**Results:**

Our study demonstrated that independent risk factors associated with the occurrence of TTP in patients with SLE included higher SLEDAI-2K score (OR = 1.96; 95%CI: 1.197–3.211; *P* = 0.007), high baseline total cholesterol (T-CHO) levels (OR = 8.19; 95%CI: 0.98–68.48; *P* = 0.048), and renal involvement (OR = 14.73; 95%CI: 1.250-173.64; *P* = 0.033). Multivariate logistic regression analysis showed that older age (OR = 1.02;95%CI: 0.94–1.119; *P* = 0.05) and non-nulliparous female (OR = 8.12; 95%CI: 0.484-136; *P* = 0.017) were independent risks factor for short-term death for SLE-TTP patients.

**Conclusion:**

SLE patients with higher SLEDAI-2K score, high baseline T-CHO levels, and renal involvement were predisposed to TTP development. The short-term mortality is increased for SLE-TTP patients of advanced age and in non-nulliparous females. Close monitoring and active treatments of these patients are needed for this life-threatening situation.

## Highlights

SLE-TTP is a severe condition with high mortality.

Renal involvement, higher baseline level of T-CHO, and high SLEDAI-2K score are risk factors for TTP development in SLE patients.

Advanced age and non-nulliparous females are risk factors for short-term death for SLE-TTP patients.

## Introduction

Systemic lupus erythematosus (SLE) is the prototype of basically all autoimmune rheumatic diseases characterized by the positivity of multiple autoantibodies and the involvement of multi-organs and multi-systems. SLE is a highly heterogeneous disease with protean manifestations and different prognoses. The disease per se and the treatments with glucocorticoids and immunosuppressants frequently cause various complications [1]. Among them, thrombotic thrombocytopenic purpura (TTP) is a life-threatening complication with a prevalence ranging from 1 to 4% [2]. TTP is one type of thrombotic microangiopathy (TMA) characterized by the combination of microangiopathic hemolytic anemia, severe thrombocytopenia, and organ impairment [3]. The loss of the activity of ADAMTS-13, an enzyme able to cleave ultra-large von Willebrand factor (vWF) multimers, is the major cause of TTP pathogenesis [4]. The autoantibodies in SLE patients can also cause endothelial damage and aberrant fibrinolysis, inciting the development of TTP [5]. TTP complicated with SLE often has a sudden onset, rapid progression, severe clinical course, and poor prognosis [6, 7]. At the early stage, TTP manifests as multi-system thrombotic microangiopathy with hemolytic anemia and thrombocytopenia, which may be accompanied by fever, neurological symptoms, renal damage, etc [8]. It is often difficult to determine when TTP is initiated during the disease course of SLE, which often leads to delayed diagnosis and treatment [9]. Early recognition of TTP in patients with SLE is critical for prompt interventions, which may prolong the survival time and reduce the mortality rate [10]. In this study, patients with SLE complicated with TTP were retrospectively analyzed to elucidate clinical and laboratory features and to explore the risk factors for TTP development. In addition to timely diagnosis, identification of risk factors responsible short-term mortality of SLE-TTP patients may help the decision-making process for aggressive treatments to improve outcomes.

## Patients and methods

This retrospective study was conducted in the First Affiliated Hospital of Zhengzhou University from 2017 to 2023. Inclusion criteria were as follows: all patients were older than 18 years; All SLE patients met the 2019 European League Against Rheumatism (EULAR) / American College of Rheumatology (ACR) classification criteria. TTP was identified based on the diagnostic criteria of the Hematology Society of the Chinese Medical Association in 2012 and the diagnostic criteria for TTP formulated by the International Society on Thrombosis and Haemostasis in 2020 [11].

The exclusion criteria were as follows: patients with other autoimmune diseases; patients accompanied with hemolytic uremic syndrome (HUS), disseminated intravascular coagulation (DIC), HELLP syndrome, Evans syndrome, and other blood-related diseases; concuruent diseases and conditions may affect the outcome, such as pregnancy and transplantation; incomplete clinical medical record data.

Data collection: The demographic and clinical characteristics were recorded, including ages, genders, childbearing history, medical history, neurologic symptoms, new rash, alopecia, mucosal ulcers, and fever. Initial and comprehensive laboratory results were collected immediately upon admission to the hospital, including blood and urine routine tests, biochemical parameters, total cholesterol (T-CHO) level, autoantibodies, and inflammatory indices. The disease activity of SLE patients was assessed by the Systemic Lupus Erythematosus Disease Activity Index 2000 (SLEDAI-2 K). Disease activity levels in these patients were designated as inactivity, mild, moderate, and severe based on their SLEDAI scores [12, 13]. All SLE-TTP patients were assessed for PLASMIC scores.

The Ethics Committee of the First Affiliated Hospital of Zhengzhou University approved this study in accordance with the Declaration of Helsinki (2024-KY-1025). Informed consent was waived by our Institutional Review Board because of the retrospective nature of our study.

### Statistical analysis

Data were analyzed using IBM SPSS Statistics (version 27.0) and R (version 4.4.1). All statistical tests were two-sided, and a *P*-value < 0.05 was considered statistically significant. According to the data distribution, continuous data were described as mean ± standard deviation (SD) or median (first quartile, third quartile). Categorical data were presented as frequencies (percentages). Statistical differences between groups were analyzed with independent samples. Student’s t-test (normal distribution), Mann-Whitney U test (non-normal distribution), and Categorical data were compared using the Chi-square test or Fisher exact test. After the stepwise regressions, collinearity diagnosis was conducted. Variables that showed a *P*-value < 0.05 in the univariate logistics were included in multivariate logistic regression to identify independent risk factors for SLE-TTP.

## Results

### Demographic features

A total of 32 SLE-TTP patients met the study criteria were included in this study, and among them, the diagnosis of SLE was made before TTP in 18 (56%) patients, while the diagnosis of SLE and TTP was simultaneously established in 14 (43%) patients. The control group (SLE without TTP) patients had a median age of 35 years (IQR, 25–44), and 95 (74%) patients were female. In SLE-TTP group, the median age was 33.5 years (IQR, 25.25–49.5), with 30 females (49%). No statistical difference was detected in age, gender, and childbearing history between these two groups.

### Clinical features

All 32 SLE-TTP had microangiopathic hemolytic anemia and thrombocytopenia, and 10 patients manifested as typical pentalogy (microangiopathic hemolytic anemia, thrombocytopenia, neurological system abnormalities, fever, and renal damage). The control group patients had a median SLEDAI-2 K score of 6 (IQR, 5–9), while the SLE-TTP patients had a median SLEDAI-2 K score of 19.5 (IQR, 17.25-22), (Table 1).

**Table 1:** Comparison of demographic features and the main clinical features between groups

Characteristics	SLE group (*n* = 128)		SLE-TTP group (*n* = 32)		
	*N* (%)	Quartile	*N* (%)	Quartile	*p*
Gender (female)	95(74.2)		30(93.8)		0.089
Age (years)		35(25–44)		33.5(25.25–49.5)	0.73
Childbearing history (pre-procreated)	81(63.3)		20(62.5)		0.905
Neurologicl symptoms	0(0)		10(31.25)		<0.001
Renal involvement	4(3.1)		26(81.3)		<0.001
New rash	70(54.7)		15(46.87)		0.482
Alopecia	22(17.2)		7(21.9)		0.559
Mucosal ulcers	11(8.6)		5(15.62)		0.444
Fever	54(42.1)		11(34.4)		0.45
SLEDAI-2 K score		6(5–9)		19.5(17.25-22)	<0.001
SLEDAI-2 K level					<0.001
Disease inactivity	31(24.2)		0(0)		
Mild disease activity	69(53.9)		1(3.1)		
Medium disease activity	25(19.5)		1(3.1)		
High disease activity	3(2.3)		30(93.8)		

Some clinical features, including neurological symptoms (0 vs. 31.25%, *P*<0.001) and renal involvement (3.1 vs. 81.3%, *P*<0.001), were more common in the SLE-TTP group than those in control group. There was no statistically significant difference in new rash, alopecia, mucosal ulcers, and fever between the two groups (*P* > 0.05).

### Laboratory findings

The levels of serum creatinine (Scr), T-CHO, CRP, and lactate dehydrogenase (LDH) in the SLE-TTP group were higher than those in control group, while the levels of Albumin (ALB) were lower than those in control group (Table 2).

**Table 2:** Comparison of laboratory findings between SLE patients with and without TTP

Characteristics	SLE group (*n* = 128)		SLE-TTP group (*n* = 32)			
	*N* (%)	Quartile	*N* (%)	Quartile	*p*	
ANA (%)					0.058	
1: 320	28(21.8)		11(34.4)			
1: 1000	46(36.0)		13(40.6)			
1: 3200	54(42.1)		8(25)			
dsDNA(IU/ml)		131.3(28.40-290.7)		164(14.85-347.63)	0.851	
Anti-Rib_P	10(7.8)		0		0.106	
Anti-PO	60(46.8)		11(34.4)		0.198	
Anti-His	41(32.0)		11(34.4)		0.718	
Anti-Nuc	60(46.8)		15(46.9)		0.81	
Anti-PCNA	3(2.3)		0		0.519	
Anti-CENP_B	3(2.3)		2(6.3)		0.251	
Anti-JO1	1(0.8)		0		1	
Anti-PMScl	0		0		1	
Anti-Scl70	2(1.5)		1(3.1)		0.481	
Anti-SSB	20(15.4)		5(15.6)		1	
Anti-Sm	50(39.1)		12(37.5)		0.968	
Anti-nRNPSm	77(60.2)		13(40.6)		0.071	
Anti-SSA	87(68.0)		19(59.4)		0.488	
Anti-Ro52	72(56.2)		17(53.1)		0.885	
Scr(µmol/L)		51(46–57)		123(61–205)	<0.001	
ALT(U/L)		17(11.25–26.5)		15(10.75–36.5)	0.815	
AST(U/L)		21(16–31)		26.5(18-43.25)	0.051	
TBIL(µmol/L)		5.815(4.7–8.31)		5.8(3.35–17.65)	0.621	
DBIL(µmol/L)		3(2.31-3.7925)		2.7(1.625-8.7)	0.944	
IBIL(µmol/L)		2.95(2.2-4.475)		2.8(1.415-12)	0.787	
ALB(g/L)		38.7(35.525–40.875)		26.3(21-30.7)	<0.001	
T-CHO(mmol/L)		3.48(2.88–4.12)		4.76(3.97–6.235)	<0.001	
ESR(mmol/h)		35(12-49.75)		45.5(17.25-69)	0.673	
CRP(mg/L)		1.95(1.465–6.69)		15.67(2.73–32.27)	<0.001	
LDH(U/L)		210(184–251)		525(292–817)	<0.001	
blood broken red blood cell count(%)				7 (6-11.75)		

No significant differences were detected in the levels of Alanine aminotransferase(ALT), Aspartate aminotransferase (AST), Total bilirubin (TBIL), Conjugated Bilirubin (DBIL), Unconjugated Bilirubin (IBIL), and erythrocyte sedimentation rate (ESR) between the two groups (all *P* > 0.05).

No statistically significant differences were detected regarding the positive rates of autoantibodies between the two groups (*P* > 0.05). In this study, all SLE-TTP patients underwent peripheral blood smear and direct antiglobulin test (DAT). A median percentage of peripheral schistocytes was 7% (IQR, 6%,11.75%), and 3 were positive for DAT (9.4%), PLASMIC scores were 6–7 in 24 (75%) cases. Serum ADAMTS13 activity was detected by FRET-VWF86 substrate assay in 10 of 32 SLE-TTP patients, all of which were significantly reduced (< 5%), while its inhibitor was detected in all of these patients.

### Risk factors for SLE-TTP

The variables causing multicollinearity were screened and eliminated by stepwise regression, which excluded collinearity among them (variance inflation factor < 3). In the univariate logistic regression, the *p*-values of these variables were below 0.05, indicating that each factor may independently serve as a risk factor for initiating TTP in SLE (Table 3).

**Table 3:** The results of univariate and multivariate logistic regression assessing the association between SLE and SLE-TTP

	Significant relapse group			Multivariate analysis		
	OR	95% CI	*p*-value	OR	95% CI	*p*-value
SLEDAI-2K score	1.989	1.532–2.582	<0.001	1.961	1.197–3.211	0.007
Neurological symptoms	146.34	48.332–799.22	0.998	-	-	-
Renal involvement	138.667	36.544-526.172	<0.001	14.732	1.250-173.64	0.033
ALB	0.727	0.651–0.812	<0.001	0.811	0.657-1.000	0.051
T-CHO	2.999	1.907–4.718	<0.001	8.191	0.98–68.48	0.048

In the stepwise regression screen after the elimination of Scr, ALB, and LDH, no collinearity was detected among them (variance inflation factor < 3). The results of the univariate analysis showed that SLEDAI-2 K score, renal involvement, ALB, and T-CHO were risk factors in the development of TTP in SLE patients. The difference was statistically significant (*P*<0.05).

As shown in Table 3, only the SLEDAI-2 K score, renal involvement, ALB, and T-CHO were included in the assessment. This adjustment accounted for interdependencies among the variables included in the model, ensuring that the identified risk factors remained robust when considering other relevant factors simultaneously.

Multivariate logistic regression analysis showed that independent risk factors for TTP development in SLE patients were as follows: SLEDAI-2 K score (OR = 1.96; 95%CI: 1.197–3.211; *P* = 0.007), T-CHO (OR = 8.19 95%CI:0.98–68.48; *P* = 0.048), and renal involvement (OR = 14.72; 95%CI: 1.250-173.641; *P* = 0.033). Moreover, the regression coefficients were all positive, suggesting that it was positively correlated with disease occurrence and was an independent risk factor for TTP in SLE patients.

### The risk of short-term death for SLE-TTP

The SLE-TTP patients were divided into two groups, those whom survived and those whom died according to the outcome of discharge, and the differences between the general data, clinical symptoms and laboratory indexes were compared between the two groups. All patients were subjected to targeted plasma exchange (infusion) and immunosuppressive therapy after diagnosis. Among them, 20 (62.5%) patients were discharged from hospital after treatment, while 12 (37.5%) patients died during hospitalization (Table 4).

**Table 4:** Comparison of baseline characteristics between survival group and death group in SLE-TTP patients

Characteristics	survival group (*n* = 20)		death group (*n* = 12)		
	*N* (%)	Quartile	*N* (%)	Quartile	*p*
Gender(female)	18(90)		12(100)		0.516
Age (years)		27.5(24-42.75)		46.5(35-57.5)	0.026
Childbearing history (*non-nulliparous*)	9(45)		11(91.7)		0.021
Neurologicl symptoms	4(20)		6(50)		0.119
Renal involvement	19(95)		7(58.3)		0.058
New rash	8(40)		7(58.3)		0.467
Alopecia	4(20)		3(25)		0.74
Mucosal ulcers	2(10)		3(25)		0.338
Arthritis	4(20)		3(25)		0.74
Myositis	8(40)		7(58.3)		0.467
SLEDAI		21(18-22.75)		18.5(16.5–23.5)	0.531
SLE duration		1(0-4.75)		2.5(1–10)	0.091
PLASMIC					0.694
5	6(30)		2(16.6)		
6	13(65)		6(50)		
7	1(5)		4(33.3)		
peripheral blood broken red blood cell count		6.5(5.25–12.25)		7(4–9)	0.481
WBC(*10^9^L)		5.585(2.905–6.9975)		3.88(2.685–5.95)	0.235
RBC(*10^12^L)		2.225(1.915–2.7175)		2.085(1.86–2.31)	0.267
Hb(g/L)		67.15(63.25–78.5)		67.35(56.75–70.75)	0.436
PLT(*10^9^L)		28(12-62.5)		32(23.5-41.75)	0.755
SII		122.652(44.5222-322.9875)		232.31(106.44-583.23)	0.199
NLR		4.145(2.6275–13.54)		7.47(3.7-13.23)	0.293
ANA (%)					0.191
1: 320	6(30)		5(41.67)		
1: 1000	7(35)		6(50)		
1: 3200	7(35)		1(8.3)		
dsDNA		298.65(10-352.475)		106.25(49.75-256.175)	0.585
Scr(µmol/L)		111.5(59.25-147.25)		157.5(93.25–264)	0.098
TBIL(µmol/L)		6.3(3.925–37.71)		4.4(2.6–5.78)	0.026
DBIL(µmol/L)		3.7(1.9-13.825)		2.25(1.6–3.36)	0.102
IBIL(µmol/L)		3.05(1.825–18.975)		2.8(0.8–4.9)	0.413
C3(g/L)		0.455(0.3125-0.66)		0.415(0.31–0.59)	0.447
C4(g/L)		0.11(0.075–0.1275)		0.105(0.08–0.12)	0.845
ESR(mmol/h)		45(9.5-71.25)		55.5(24–68)	0.907
CRP(mg/L)		32.18(10.46–32.27)		3.045(2.39–17.39)	0.051
CK(U/L)		47(33.5–180)		80.5(34-128.5)	0.741
ckmbo(U/L)		14.15(11.63–22.68)		16(11–25)	0.953
LDH(U/L)		510.5(264–707)		669(508–895)	0.102
HBDH(U/L)		324.5(236-654.7)		551.5(345–866)	0.124
LA	2(10)		2(16.7)		0.227
ACL	3(15)		0(0)		0.274
β2-GP1	4(20)		2(16.7)		0.815
infection	13		11		0.092
therapy					
Plasma exchange+immunosuppressive agent	20(100)		12(100)		-
Super-high-dose GCs	14		8		0.844
rituximab, RTX	5		1		0.37
IVIG pulse therapy	9		5		0.854

To identify differences in laboratory indices that predict short-term survival status in patients with SLE-TTP, we analyzed the baseline data between the two groups and found significant differences in patient ages, childbearing history, and baseline TBIL level.

There were no significant differences in gender, neurological symptoms, renal involvement, SLEDAI-2 K, PLASMIC score, direct antiglobulin test (DAT), ANA titer, and the concentrations of complements between the survival group and death group. In the multiple logistic regression analysis, the risk factors with *P* < 0.05 in the single-factor logistics analysis were included. Multivariate unconditional logistic regression showed that older age (OR = 1.02,6–95%CI: 0.94–1.119; *P* = 0.05) and non-nulliparous female (OR = 8.12, 95%CI: 0.484-136; *P* = 0.017) were associated with the risk of short-term death for SLE-TTP patients (Table 5).

**Table 5:** The results of univariate and multivariate logistic regression assessing the association between survival group and death group in SLE-TTP patients

	Significant relapse group			Multivariate analysis		
	OR	95% CI	*p*-value	OR	95% CI	*p*-value
Age (years)	1.08	1.01–1.149	0.025	1.02	0.94–1.119	0.05
Childbearing history(non-nulliparous)	13.44	1.448-124.859	0.022	8.12	0.484-136	0.017
TBIL(µmol/L)	0.84	0.665–1.073	0.166	-	-	-

## Discussion

TTP is a rare but fatal condition characterized by microangiopathic anemia and thrombocytopenia largely due to the deficiency or decreased activity of ADAMTS13 [14]. TTP can be induced by severe infections, severe hepatitis, tumors, autoimmune diseases, pregnancy, and certain medications [15]. However, classic pentad is not commonly seen in clinical practice, and it only appears only in < 7% of the patients at the onset of the disease. The protean clinical manifestations make it difficult to diagnose TTP. In addition, the development of TTP in SLE patients is rare, and these two diseases have some overlapping clinical features, which makes the diagnosis even more difficult [16]. TTP is a life-threatening condition with a mortality up to 90% if left untreated, whereas early detection and prompt treatment may reduce the mortality to 9-21% [17, 18]. Our study aimed to reveal the risk factors for TTP development through analysis of clinical features for early intervention.

Multivariate logistic regression analysis showed that independent predictors for TTP development in SLE patients were SLEDAI-2 K score, T-CHO and renal involvement (*P*<0.05). SLE patients with high baseline level of T-CHO, higher SLEDAI-2K score, and renal involvement were predisposed to TTP development. Consistent with our results, previous studies have shown that high SLEDAI scores may, to some extent, be held responsible for the development of TTP in SLE patients [19, 20]. Of note, no significant differences were detected in the positive rates of ANA, anti-dsDNA, and other autoantibodies between the two groups. The most important finding in our study is that the SLE-TTP group has higher T-CHO, which may lend some novel insights into SLE-TTP pathogenesis although the detailed mechanism is not fully understood. Chung, et al. have shown that elevated levels of LDL trigger vWF self-association into fibers and bundles and potentiate microvascular thrombosis [21]. On the one hand, it is considered that it is related to the stimulation of excessive liver synthesis caused by hypoalbuminemia. Further, it is likely that hyperlipidemia per se may induce the formation of TTP.

Neurologic symptoms were considered as independent risk factors for potential TTP development in SLE patients. However, our results show that neurological involvement is not an independent risk factor for the formation of TTP in SLE. An additional finding in our study, was that ALB is a protective factor in the occurrence of SLE-TTP. When unexplained anemia and thrombocytopenia are present in SLE patients, a peripheral blood smear should be done immediately and repeatedly. If more than 1% schistocytes are detected on the blood smear, it would indicate potential thrombotic microangiopath [22]. Schistocytes and ischemic tissue necrosis may increase serum levels of LDH, which may serve as an indicator of disease severity and TTP development.

Our results show that older age is an independent risk factor for short-term death of SLE-TTP patients, consistent with a previous report [23]. In addition, we demonstrate that women who have a child-birth history are predisposed to TTP development. A reasonable explanation is that the pathogenesis and progression of TTP in SLE patients may be associated with hormonal changes and pregnancy-related complications, and special attention should be paid to these patients.

Previous studies have shown that infection may be an important factor for TTP formation in SLE patients [20], however, we did not find significant differences between the survival group and the death group regarding infection (*P* > 0.05). All patient were treated with plasma exchange (infusion) and immunosuppressants after diagnosis. No statistically significant differences were found between the two groups with regard to additional treatments such as glucocorticoids(GCs), intravenous immunoglobulin (IVIG), and rituximab.

Although the detailed mechanism of TTP development in SLE remains largely unknown, recent studies indicate that TTP and SLE share some common features in terms of genes, signaling pathways, and molecular signatures [24, 25]. The autoantibodies against the depolymerized protein-like ADAMTS-13 containing the platelet-binding protein motif of type I in SLE patients may reduce the activity of ADAMTS13 with subsequent impairment of cleavage of vWF, leading to the formation of microthrombus together with platelets [26–28].

In regard to the limitations of our study, due to the low incidence, the sample size of this study is not large enough. In addition, only a portion of the SLE-TTP patients tested the activity of ADAMTS13 and the levels of its inhibitor. Furthermore, a possible partial selection bias may increase the sampling error. Other confounding factors such as medications and comorbidities may also affect the interpretation of the results. Therefore, more randomized controlled studies with larger samples are needed to further verify the predictive value of these indicators for development of TTP.

## Conclusions

SLE patients with high baseline level of T-CHO, higher SLEDAI-2K score, and renal involvement are predisposed to TTP development. Close monitoring of these risk factors is needed for early identification and prompt intervention of this fatal situation to reduce the mortality. Special attention should be paid to SLE-TTP patients who are of advanced age and/or non-nulliparous females as they have high short-term mortality. There should be consideration of aggressive early intervention to reduce mortality in this high risk group.

## Data Availability

The data are available from the corresponding author upon reasonable request.
